# Levels of Alpha-Fetoprotein and Association with Mortality in Hepatocellular Carcinoma of HIV-1-Infected Patients

**DOI:** 10.1155/2022/3586064

**Published:** 2022-02-10

**Authors:** Giulia Morsica, Laura Galli, Emanuela Messina, Alessandro Cibarelli, Sabrina Bagaglio, Andrea Poli, Martina Ranzenigo, Antonella Castagna, Hamid Hasson, Caterina Uberti-Foppa

**Affiliations:** ^1^Dept. of Infectious Diseases, San Raffaele Hospital, Milan, Italy; ^2^Vita-Salute University, Milan, Italy

## Abstract

**Methods:**

This is a retrospective cohort study on patients living with HIV-1 infection (PLWH) followed at the Division of Infectious Diseases of the San Raffaele Hospital, with cirrhosis and HCC diagnosed between 1999 and 2018 and with an available AFP value at HCC diagnosis. The area under the receiver operating characteristic curve (AUC) was used to estimate the accuracy of baseline AFP in predicting death. Factors associated with the risk of death were identified using multivariable Cox proportional-hazards regression models.

**Results:**

Overall, 53 PLWH were evaluated: 18 patients received a curative treatment (9 liver transplantation, 5 liver resections and 4 radiofrequency ablation) and 35 a noncurative treatment (17 chemo or radio embolization, 10 sorafenib and 8 best supportive care). Baseline AFP was predictive of death [AUC 0.71, 95% confidence interval (CI) 0.54–0.83], and the optimal cut-off was 28.8 ng/mL. At multivariable analysis, BL AFP ≥28.8 ng/mL was associated with death [adjusted hazard ratio (aHR) 7.05, 95% CI 1.94–25.71 *P* = 0.003]. Other factors were HBV infection (aHR 8.57, 95% CI 1.47–50.08, *P* = 0.017) and treatment allocation (curative vs. noncurative, aHR 0.08, 95% CI 0.02–0.40, *P* = 0.0004).

**Conclusions:**

Our findings suggest that in PLWH AFP serum levels ≥28.8 ng/mL, HBV coinfection and treatment allocation represent predictive markers for death at the time of HCC diagnosis.

## 1. Introduction

Hepatocellular carcinoma (HCC) is the fourth most common cause of death worldwide.

The high prevalence of coinfection with hepatitis B virus (HBV) and hepatitis C virus (HCV), the hepatotoxicity associated with antiretroviral therapy and alcohol abuse has made HCC a rapidly increasing cause of morbidity and mortality, currently accounting for up to 50% of liver-related deaths in PLWH [1–3].

Nevertheless, the clinical course of HCC in an HIV-1-infected setting is not well defined yet, probably because of the heterogeneity in the characteristics of the studied groups, such as, for instance, differences in HCC aetiology, use of ART, and use of anticancer therapy.

It has been shown that alpha-fetoprotein (AFP) levels correlate with mortality in patients with HCC in the nontransplant setting, across all aetiologies, and specifically in patients with chronic HCV infection [4]. Alpha-fetoprotein is therefore incorporated into several prognostic scoring systems [the Cancer of the Liver Italian Program (CLIP), the Chinese University Prognostic Index (CUPI), Groupe d'Etude et de Traitement du Carcinoma Hépatocellulaire (GRETCH), and French scoring system AFP model [5].

It remains controversial if AFP can be used as an independent prognostic factor, with some authors believing that AFP is a prognostic marker affecting the long-term survival of people with HCC.

The actual prognosis effect of AFP incorporated into a score or as independent marker is either unclear or limited in the PLWH population.

Given that AFP is an inexpensive, simple, reliable, and widely available tool, it is important to examine the prognostic properties of AFP in an HIV-1-infected setting. Therefore, we decided to evaluate the effect of serum AFP at HCC diagnosis on survival in PLWH and to determine the factors associated with the risk of death.

## 2. Methods

### 2.1. Study Population

This is a retrospective cohort study on 84 PLWH with incident HCC diagnosed between November 1999 and May 2018; of these 84 PLWH, 53 had alpha-fetoprotein levels available at HCC diagnosis (baseline (BL) evaluation).

Data were collected as part of routine clinical care and recorded in the database of the Division of Infectious Diseases of the San Raffaele Hospital (CSLHIV Cohort). The CSLHIV Cohort was approved by the ethics committee of the San Raffaele Hospital, Milan, Italy. On their first visit, patients provide written informed consent on the use of their data in scientific analyses. Recorded data are anonymized and managed according to the Good Clinical Practice.

Serum AFP levels were required within 60 days of HCC diagnosis. All the other data were also collected at the time of HCC diagnosis. HCC diagnosis was based on imaging or on histologic criteria according to international guidelines [6].

The individuals were staged according to Child–Turcotte–Pugh (CTP) functional class and Barcelona clinic liver cancer (BCLC) algorithm following standard prepublished methodology [7, 8].

Treatment was differentiated in curative (liver transplantation, LT; liver resection LR; radiofrequency ablation, RABL) and noncurative (chemo or radioembolization, CRE; sorafenib, SOR; best supportive care, BSC). Patients who underwent more than one type of treatment were categorized according to the following hierarchy: LT, LR, RABL, CRE, SOR, and BSC.

In the analyses, the main exposure variable of interest was serum AFP levels at HCC diagnosis. The main outcome variable was time to death after HCC diagnosis.

### 2.2. Statistical Analysis

Results were expressed as median (interquartile range, IQR) or frequency (%). Continuous variables were compared using the Wilcoxon rank-sum test; categorical variables were compared using the chi-square or the Fisher's exact test, as appropriate. Imputation for missing data was not performed.

The ability of baseline AFP in predicting death was determined by the area under the curve (AUC) of receiver operating characteristics (ROC) curves. The optimal cut-off value, predicting the presence or absence of death, was determined on the highest Youden index value (sensitivity + specificity-1). The diagnostic accuracy of this parameter was evaluated in terms of sensitivity, specificity, negative predictive value, and positive predictive value with the corresponding 95% confidence intervals (95% CIs) either at the optimal cut-off or at the 200 ng/mL cut-off levels according to EASL guidelines for HCC management [6]. The 400 ng/mL cut-off recommended in the international guidelines as associated with worse prognosis of HCC was not considered because 79.2% of our patients had BL AFP levels lower than 400 ng/mL.

Total accuracy was also assessed by the percentage of patients that were correctly classified according to the estimated optimal cut-off.

Kaplan–Meier curves were calculated to estimate time to death according to baseline AFP (stratified on the optimal cut-off or the 200 ng/mL cut-off levels); curves were compared by the log-rank test.

Two multivariable Cox proportional-hazards models were applied to compare strata of baseline AFP (stratified on the two different cut-offs: ROC cut-off and the 200 ng/mL, respectively) with respect to death. Models included also tumour-related factors, treatment allocation, hepatic reserve, and clinical parameters related to the severity of HIV infection; the choice of covariates was based on current knowledge on factors with a suspected or an established effect on death.

The results of these analyses were reported as adjusted hazard ratios (aHR) with the corresponding 95% confidence intervals (CIs). The assumption of the proportional hazards was examined.

Two-sided *P* values were considered statistically significant if less than 0.05. Statistical analyses were performed using SAS Software, release 9.4 (SAS Institute, Cary, NC).

## 3. Results

### 3.1. Patient's Characteristics

The baseline characteristics of the 53 individuals included in this study are summarized in Table 1. There was a preponderance of male gender (*n* = 45 (85%)), and the main risk factor for HIV-1 infection was past or actual drug use (*n* = 26 (49%)).

At baseline, HIV viral load was suppressed (<50 copies/mL) in 43 (81%) PLWH.

The majority of PLWH were hepatitis C carriers (36 (68%)) with HCV genotype (GT) available in 23 patients: GT3a was the predominant infecting virus (13/23, 57%), and the other GT were 1a in 5 patients, 1b in 2 patients, and 4 in 3 patients. Of these 36 patients with HCV infection, 12 were treated for chronic hepatitis C before HCC diagnosis: 8 received interferon-ribavirin with no response in 4 of them, while the remaining 4 patients received direct acting antivirals (DAAs) with sustained virological response.

Of 17 patients with hepatitis B virus (HBV) infection, 3 patients had concomitant HCV and one coinfection with hepatitis Delta virus. Fourteen/17 (82%) HBV-infected patients were under treatment with nucleot(s)ide analogs with undetectable HBV-DNA levels (limit of detection 10 IU/mL).

Child–Turcotte–Pugh (CTP) score was A in 40 (75.5%) PLWH, B in 9 (17%), and C in 4 (7.5%); BCLC stage was 0/A in 18 (34%) PLWH, B in 11 (21%), and C/D in 24 (45%) PLWH. The treatment allocation with regard to BCLC stage is summarized in Table 2. Eighteen PLWH (34%) received a curative treatment [9 LT, 5 LR, 4 RABL] and 35 PLWH a noncurative treatment: 17 CRE, 10 SOR, and 8 BSC. Nonadherence to the original BCLC treatment recommendation was observed in 28 (52.8%) HCC patients. Twenty HCC patients received upward treatment stage migration, while 8 patients received downward treatment stage migration.

In particular, 6 patients with terminal BCLC stage received sorafenib: one patient (CTP stage B) stopped treatment for liver decompensation, 3 patients (2 in CTP B and one CT P C) discontinued treatment for HCC progression, 1 patient (CTP C) was lost to follow-up, and the remaining 1 patient with CTP C stopped sorafenib for toxicity (thrombocytopenia).

Overall, median AFP value was 41.8 ng/mL (IQR 15.1–256.0), while, according to BCLC stage, median AFP levels were 24.5 ng/mL (IQR 8.3–174.3) in patients with very early/early stage, 45.7 ng/mL (19.4–347.7) in patients with intermediate/advanced stage, and 40.3 ng/mL (IQR 27.8–367.9) in patients with terminal stage.

### 3.2. Baseline AFP Levels

**Figure 1:**
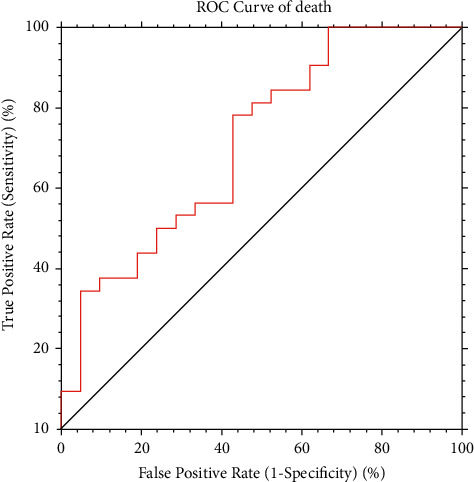
Diagnostic performance and optimal cut-off value of baseline AFP for hepatocellular carcinoma prognosis (death). ROC : receiver operating characteristics of the curves; AUC : area under the curve; PPV : positive predictive value; NPV : negative predictive value.

Baseline AFP values ≥28.8 ng/mL were deemed the best performing for identifying high-risk people for death (sensitivity = 78%, specificity = 57%, see ROC curves in Figure 1).

The logistic model correctly classified 69.8% of individuals and the model discrimination was good (AUROC = 0.71 [0.54, 0.83]).

Characteristics of patients according to the estimated BL AFP cut-off are summarized in Table 1.

Baseline AFP was ≥28.8 ng/mL in 34 (64%) PLWH. The median values of BL AFP were 136 ng/mL (IQR 45–645) in patients with AFP ≥28 ng/mL and 11.3 ng/mL (IQR 6.9–18.3) in those with AFP <28/ng/mL.

The HCC patients with BL AFP ≥28 ng/mL had more frequently a multinodular disease with respect to those with BL AFP <28 ng/mL (*P* = 0.006). Pseudocholinesterase value was found significantly lower in patients with AFP ≥28 ng/mL, with respect to those with AFP< 28 ng/mL (*P* = 0.016). Total bilirubin levels were slightly higher in patients with AFP ≥28 ng/mL, with a trend toward significance (*P* = 0.09).

The HCC patients with BL AFP ≥28 ng/mL less frequently received a curative treatment for HCC with respect to those with AFP <28 ng/mL (24% vs. 53% *P* = 0.039). The other variables attaining tumor-related factors, hepatic reserve, and clinical parameters related to the severity of HIV infection were similarly distributed in patients with AFP ≥28 or <28 ng/mL.

Characteristics of patients according to BL AFP ≤200 ng/mL, or >200 ng/mL, are summarized in Table 3. Fifteen (28.3%) patients had a BL AFP value >200 ng/mL. The median value of BL AFP was 821 ng/ml (IQR 368–3458) among patients with BL AFP >200 ng/mL and 28.3 ng/mL (IQR 11.3–46.0) among patients with BL AFP ≤200 ng/mL.

The intrahepatic burden of disease assessed by the number and size of lesions was similar in patients with AFP >200 or ≤200 ng/mL. Patients with AFP >200 ng/mL had a shorter median follow-up (0.79 years (IQR 0.43–1.1)) compared to those with AFP ≤200 ng/mL (2.57 years (IQR 0.67–4.95); *P* = 0.008). The presence of extrahepatic metastasis tended to be higher in patients with AFP >200 ng/mL, showing a trend toward significance (*P* = 0.084).

HCC patients with BL AFP >200 ng/mL less frequently received a curative treatment with respect to patients with BL AFP ≤200 ng/mL (13% vs. 42%, *P* = 0.046). The other variables were similarly distributed in patients with AFP > or ≤200 ng/mL.

### 3.3. Death Probability

During a median follow-up of 20 months (IQR = 8–42), 32 (60.4%) PLWH died. Overall, the 1- and 2-year cumulative probabilities of death were 30.2% (95% CI 19.4%–45.1%) and 47.4% (95% CI 34.4%–62.3%), respectively. According to the estimated BL AFP cut-off, 25 (74%) deaths occurred among patients with BL AFP ≥28 ng/mL compared to 7 (37%) with BL AFP <28 ng/mL (*P* = 0.008); 12 (80%) deaths occurred among patients with BL AFP ≥200 ng/mL compared to 20 (53%) with BL AFP ≤200 ng/mL (*P* = 0.067).

**Figure 2:**
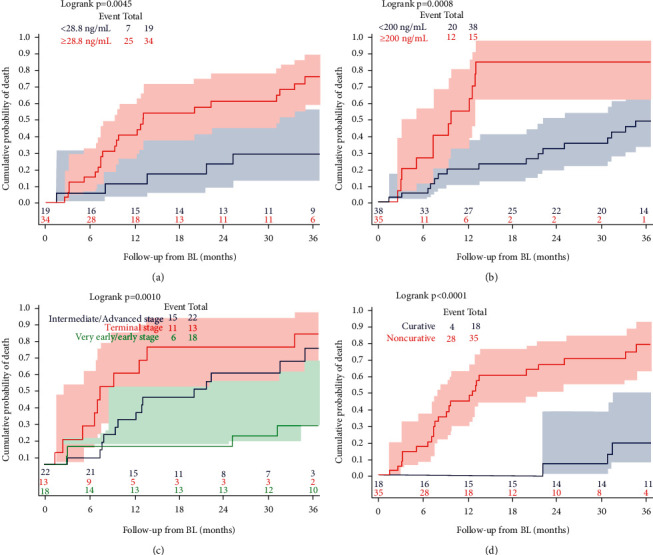
Cumulative probability of death in HIV-1 patients with hepatocellular carcinoma according to baseline AFP (at the best cut-off of 28.8 ng/mL, panel (a) or at 200 ng/mL, panel (b)); BCLC stage (panel (c); very early/early stage; intermediate/advanced stage; terminal stage) and type of HCC treatment (panel (d); curative vs. noncurative).

The probability of death in relation to the BL AFP cut-offs of 28.8 ng/mL and 200 ng/mL is displayed in Figures 2(a) and 2(b).

Time to death was significantly different between PLWH with BL AFP <28 and ≥28 ng/mL (log-rank test: *P* = 0.005). The 1-year cumulative probability of death was 11.2% (95% CI 2.9%–37.9%) among PLWH with BL AFP <28.8 ng/mL and 40.6% (95% CI 25.9%–59.5%) among those with BL AFP ≥28.8 ng/mL; at 2 years, the probabilities of death were 23% (95% CI 9.3%–50.4%) vs. 60.9% (95% CI 44.3%–77.8%) among individuals with BL AFP <28 and ≥28 ng/mL, respectively.

Twelve (80%) deaths occurred among patients with BL AFP ≥200 ng/mL compared to 20 (53%) with BL AFP ≤200 ng/mL (*P* = 0.067).

Time to death was significantly different even when considering the cut-off of 200 ng/mL for BL AFP (log-rank test: *P* = 0.0008): the 1-year cumulative probability of death was 20.0% (95% CI 10.0%–37.4%) vs. 55.0% (95% CI 32.2%–80.6%), respectively; at 2 years, the estimated probability was 32.2% (95% CI 19.2%–50.6%) vs. 85% (95% CI 62.2%–97.5%), among people with BL AFP ≤200 ng/mL or >200 ng/mL, respectively.

Figure 2(c) shows the cumulative probability of death according to BCLC stage: the best prognosis was estimated for patients with very early/early stage, while the worse prognosis was observed in those with terminal BCLC stage (log-rank test: *P* = 0.001).

The cumulative probability of death according to the use of a curative treatment is shown in Figure 2(d): patients who received a curative treatment had a much more favourable prognosis than those who received a noncurative treatment or best supportive care (log-rank test: *P* < 0.0001).

By multivariable analyses, in the model 1, BL AFP at the optimal cut-off of 28 ng/mL was a prominent factor associated with death (Table 4). The hazard ratio for death was 7.05, 95% CI 1.94–25.71, *P* = 0.003 with BL AFP ≥28.8 as compared with BL AFP <28.8. Other predictive factors were HBV coinfection and type of HCC treatment (curative vs. noncurative (Table 4)).

In the model 2, BL AFP at value >200 ng/mL was associated with death; the hazard ratio was 3.14 95% CI 1.11–8.88, *P* = 0.031. One other predictive factor was the type of HCC treatment (Table 4).

## 4. Discussion

The prognostic significance of AFP has been established in HCC patients with varied risk magnitudes depending on the defined AFP cut-offs [9–11]. However, in the majority of these reports, AFP levels have been incorporated into prognostic score system, rather than considered as a single prognostic marker.

One study performed in HIV-1-negative patients showed that AFP value higher 10 ng/mL was associated with poor survival in HCC patients after liver resection [12].

The study by Toader et al. [13] showed a correlation between tumor size, as well as number of nodules, portal thrombosis, and elevated AFP protein (>200 ng/mL). However, this study did not investigate the significance of AFP levels as possible prognostic factor for survival in patients with HCC.

One other study performed in HIV-negative patients [14] showed that AFP≥ 400 ng/mL is a reliable tool in the prognosis of HCC patients: HCC patients with a AFP concentration ≥400 ng/mL tend to have greater tumour size, massive or diffuse types, portal vein thrombosis, and a lower median survival rate.

In the report by Duvoux et al. [15], the AFP level at listing was an independent predictor of post-transplantation survival for HCC in HIV-uninfected patients.

A recent study [16] evaluating the impact of HIV infection on HCC outcome showed that HIV-1-infected patients with high AFP values had a 18% greater risk of death and that functional reserve assessed by CTP class (A vs. C). was associated with poor survival.

To the best of our knowledge, this is the first study showing that AFP levels at a well-defined cut-off is an independent risk factor for death among PLWH with HCC.

Since the AFP cut-off predictive of survival was lower than those reported in previous studies performed in HIV-negative patients [10, 14, 17], we could hypothesize that HCC arised in the concomitant HIV infection could be biologically different from other aetiologies. In this regard, we found that patients with AFP ≥28.8 ng/mL had more frequently a multinodular disease with respect to those with AFP <28 ng/mL, whereas using the cut-off of 200 ng/mL, we did not find an association with the presence of multinodular disease in our group of PLWH (see Tables 1 and 2).

A number of studies investigating the clinical significance of AFP in HCC [12, 18, 19] showed that AFP is a key molecule involved in proliferation, angiogenesis, and apoptosis. Additionally, it seems to elicit the escape of carcinoma cells from the immune surveillance. Since HIV-1 induces “per se” immune dysregulation, AFP could negatively impact the clinical outcome of HCC in HIV-1-infected patients at lower levels, with respect to those detected in the HIV-negative counterpart.

One other explanation for this our finding, is that the low cut-off value for AFP we found associated with HCC prognosis, reflects a trend for early detection of HCC. The cut-off value of 28.8 ng/mL was obtained in a group of HCC patients where 34% of subjects received a curative treatment. Notably, the treatment allocation (curative vs. non curative) was associated in the multivariate analysis with the risk of death and 47.4% of patients with AFP <28.8 ng/mL had BCLC 0/A, while 26.5% of patients with AFP ≥28.8 ng/mL had BCLC 0/A. Altogether, these our findings suggest that a number of patients with AFP <28.8 ng/mL may have HCC at early stage.

We showed that HBV infection but not HCV was associated with worse survival, while none of the parameters indicative of the severity of HIV disease (CD4 T cells nadir, CD4 T cells count, and HIV-RNA load) was revealed as predictors of poor survival, probably as a consequence of good HIV control in our group of patients. The finding of a negative effect of HBV infection on survival in our series of PLWH with HCC is in line with previous reports [20, 21] indicating that HIV/HBV coinfection seems to accelerate the progression of liver diseases with respect to HBV monoinfection, leading to increased risk of cirrhosis and possibly liver cancer.

In our group of PLWH, the survival during a median follow-up of 20 months was 60.4%.

Overall, the 1- and 2-year cumulative probabilities of death were 30.2% and 47.4%, respectively.

Although we did not compare PLWH with a group of PNLWH, we showed that the survival in PLWH is similar to that of HIV-negative individuals, [22–24] when they receive appropriate anticancer treatment.

Studies comparing groups matched for HIV-1 status reported a significantly shorter survival in HIV-1-infected patients with respect to HIV-1-negative patients. In the report by Puoti et al. [25], 60% of the HIV-positive patients did not receive anticancer treatment, and the majority of their patients had multinodular or infiltrating HCC. More recently, Pinato et al. [16] investigated the impact of HIV seropositivity on HCC outcome, and showed that HIV adversely influences the clinical behaviour of HCC. The median survival was 2.2 months in PLWH. In contrast, we showed that after 20 months, 60.4% of HCC patients were alive. We had 45.3% of HIV-1 positive patients with intermediate-advanced stage of HCC according to BCLC classification, while in the study by Pinato et al. 85.6% of HIV-1-infected patients had intermediate-advanced HCC stage.

Therefore, the different characteristics of patients as well as clinical end point between our study and that by Pinato are likely responsible for the discrepancy in the survival rate.

In the study by Merchante et al., [26] 1- and 3-year survival was 50% and 31% in PLWH and 69% and 34% in those without HIV (*P* = 0.16) with 33% of HIV-infected patients showing BCLC = 0/A vs. 56% of HIV uninfected. In our group of PLWH, we found a higher cumulative probability of survival at one year (68%) with a similar prevalence of BCLC = 0/A (34%).

Brau et al. [27] showed similar survival in patients living (6.9 months) with HIV or not (7.5 months, *P* = 0.44) with BCLC stages C or D present in 50% of HIV-positive and in 58.4% of HIV-negative patients. Median survival was also no different between HIV-positive and HIV-negative subjects when separating those who did receive potentially curative therapy (17 vs. 22 months, *P* = 0.14) and those who did not (5.5 vs. 4.4 months, *P* = 0.98).

Interestingly, the multicentre study by Berretta et al., [28] which retrospectively evaluated survival of 97 HIV-1-positive and 338 HIV-1-negative HCC patients, revealed worse prognosis in HIV-1-infected patients including those who had or not treatment for HCC, with respect to the counterpart of HIV-uninfected patients. The median survival in their HIV-positive patients was 35 months, which is a longer period with respect to that we found in the present study. However, the report by Berretta et al. [28] included a higher number of patients with curative treatment (44.9%) with respect to those in our study (34%). Additionally, we had a higher number of patients with advanced-terminal BCLC stage comparing our groups of patients (45.3%) with those (33.6%) in the study by Berretta et al. [28].

So, the different characteristics of study population, regarding BCLC stage and treatment allocation, may be responsible for discrepancy in survival.

Nonadherence to the original BCLC guidelines [29, 30] is common in real-world practice. In this regard, a subset of BCLC intermediate and advanced-stage patients underwent a more aggressive treatment than what were recommended by the BCLC system. In particular, 7 patients in BCLC stage C and 3 in BCLC stage D received CRE, 6 patients in stage D were treated with sorafenib, and one HIV/HBV-coinfected patient in BCLC stage D and CTP C was transplanted in year 2012 and is still alive.

Although most current guidelines recommend sorafenib for CTP A patients, in real-world practice also CTP B patients are frequently treated with sorafenib. In this regard, a recent report investigating the benefit of sorafenib in patients with HCC and CTP B cirrhosis [31] suggested that treatment with sorafenib should be considered in a selected subset of CTP B population.

Only one previous study on sorafenib treatment in PLWH [32] evaluated also patients with CTP C, showing liver decompensation during treatment in 100% of patients with CTP C.

In the present study, of 6 patients with BCLC stage D who received sorafenib, 3 were on CTP B and 3 on CTP C: only one of these 6 patients stopped treatment for decompensated cirrhosis.

Therefore, our findings suggested that HIV-positive patients with HCC could be treated with a more aggressive approach with respect to that proposed by the BCLC staging algorithm.

Notably, the median survival in our patients under noncurative treatment was 12.8 months (see Figure 2). This our result is similar to that reported in other studies on HIV-infected patients with noncurative treatment [25, 27].

Our study acknowledges a number of limitations. First, this study design is retrospective, which might be associated with misclassification or information bias; although data collection was not specifically planned for this study, the CSLHIV Cohort database prospectively collects many detailed information on all the individuals followed in our centre, so that we are confident that such biases are limited and our data are reliable. Second, as the study was conducted in a single centre, the generalizability of our findings may be limited; however, a single-centre study is more homogeneous with respect to multicohort studies in term of access to care, availability, and adherence to surveillance programms, anticancer treatment, and quality of palliative care support after HCC diagnosis. Third, although AFP levels seem to affect survival, the study might not be sufficiently powered for this outcome due to the small sample size.

Another limitation relies on the lack of a control group; we tried to overcome this limitation by comparing our outcomes with historical studies on PLWH or on HIV-1-negative individuals with similar or different characteristics.

In conclusion, we showed that AFP levels at a precise cut-off at HCC diagnosis are predictive of subsequent mortality in PLWH. Other related factors were viral aetiology (HBV) found at the basis of HCC, as well as the type of HCC treatment, but not parameters related to the severity of HIV infection.

Testing for AFP at HCC diagnosis is inexpensive, widely available, easily interpretable, and may help to clarify prognosis. We believe that it could be of clinical utility to incorporate AFP levels at a precise cut-off in a staging system including also aetiology at the time of HCC diagnosis and that such system could be relevant for the management of patients with HIV-associated HCC.

Future studies on a larger group of PLWH with HCC are needed to better validate our conclusions.

## Figures and Tables

**Table 1:** Characteristics at HCC diagnosis of PLWH in the overall sample and according to baseline alphaphetoprotein (cut-off = 28.8 ng/mL).

Characteristics	Category	Overall (*n* = 53)	BL AFP ≥28.8 ng/mL (*n* = 34)	BL AFP <28.8 ng/mL (*n* = 19)	*P* value
Age		53 (48–56)	53 (48–55)	54 (50–58)	0.388
Male sex (%)		45 (85)	28 (82)	17 (90)	0.696
Risk factor for HIV (%)	Drug users	26 (49)	18 (53)	8 (42)	0.727
	Sexual exposure	11 (21)	7 (21)	4 (21)	
	Other/Unknown	16 (30)	9 (26)	7 (37)	

Years of HIV infection		24.2 (16.3–27.2)	24.7 (18.5–27.5)	20.0 (12.7–26.4)	0.287
Years of ART		14.7 (9.5–17.8)	15.1 (10.0–17.8)	11.2 (7.8–18.8)	0.388
CD4 nadir cells count, n/*μ*L		105 (59–229)	105 (52–236)	116 (64–229)	0.613
CD4 cells count, *n*/*μ*L		392 (222–598)	384 (207–613)	403 (260–583)	0.690
CD8 cells count, *n*/*μ*L		626 (391–1115)	552 (389–902)	824 (445–1392)	0.426
CD4/CD8 ratio		0.53 (0.34–1.09)	0.64 (0.34–1.13)	0.42 (0.31–0.71)	0.333

Anti-HCV (%)	Positive	36 (68)	25 (74)	11 (58)	0.372
	Negative	14 (26)	8 (24)	6 (32)	
	Unknown	3 (6)	1 (2)	2 (10)	

HBsAg (%)	Positive	17 (32)	9 (26)	8 (42)	0.200
	Negative	33 (62)	24 (71)	9 (47)	
	Unknown	3 (6)	1 (3)	2 (11)	

Calendar year HCC diagnosis		2012 (2010–2014)	2012 (2010–2014)	2012 (2008–2015)	0.675
AFP, ng/mL		41.8 (15.1–256)	135.5 (45.3–645)	11.3 (6.9–18.3)	<0.0001
AST, IU/L		71 (39–111)	88.5 (43.5–115.5)	56 (31–96)	0.166
ALT, IU/L		67 (31–100)	69 (32–101)	67 (27–87)	0.659
Pseudocholinesterase IU/L		4500 (2.800–6510)	3760 (2570–5010)	5980 (4870–7910)	0.016
Total bilirubin, mg/dL		1.09 (0.80–1.85)	1.29 (0.97–2.32)	1.03 (0.78–1.35)	0.099

Child–Turcotte–Pugh score (%)	A	40 (75)	25 (73)	15 (79)	0.277
	B	9 (17)	6 (18)	3 (16)	
	C	4 (8)	3 (9)	1 (5)	

Nodules number (%)					
	1	18 (34)	9 (26)	9 (47)	0.006
	2	6 (11)	1 (3)	5 (26)	
	3	4 (8)	4 (12)	0 (0)	
	>3	25 (47)	20 (59)	5 (26)	
	<3	24 (45)	10 (29)	14 (74)	0.004
	>3	29 (55)	24 (71)	5 (26)	

Maximum tumor size, cm		3.0 (2.2–5.5)	3.0 (2.1–4.0)	2.7 (2.0–8)	0.888
Presence of portal vein thrombosis (%)		19 (36)	12 (35)	7 (37)	0.910

Extrahepatic metastasis (%)	Yes	4 (8)	3 (9)	1 (5)	0.664
	No	48 (91)	30 (88)	18 (95)	
	Unknown	1 (1)	1 (3)	0	

BCLC stage (%)	0	9 (17)	3 (9)	6 (32)	0.250
	A	9 (17)	6 (18)	3 (16)	
	B	11 (21)	9 (27)	2 (11)	
	C	11 (21)	7 (21)	4 (21)	
	D	13 (25)	9 (27)	4 (21)	

HCC treatment (%)	Curative	18 (34)	8 (24)	10 (53)	0.039
	LR	5	3	2	
	LT	9	5	4	
	RABL	4	0	4	
	Noncurative	35 (66)	26 (77)	9 (47)	
	CRE	17	12	5	
	SOR	10	7	3	
	BSC	8	7	1	

Results are described by median (IQR) or frequency (%). BL : baseline; AFP : alphaphetoprotein; ART : antiretroviral therapy; *n* : number; HBsAg : hepatitis B surface antigen; AST : aspartate aminotransferase (normal values <35 IU/L); ALT : alanine aminotransferase (normal values <59 IU/L); BCLC: Barcelona Clinic Liver Cancer; LR : liver resection; LT : liver transplantation; RABL : radiofrequency ablation; CRE : chemo or radioembolization; SOR : sorafenib; BSC : best supportive care.

**Table 2:** BCLC stage and treatment for hepatocellular carcinoma in PLWH.

BCLC stage	LT (*n* = 9)	LR (*n* = 5)	RABL (*n* = 4)	CRE (*n* = 17)	SOR (*n* = 10)	BSC (*n* = 8)	Total (*n* = 53)
0/A (%)	8 (89)	2 (40)	4 (100)	2 (12)	1 (10)	1 (13)	18 (34)
B (%)	0 (0)	2 (40)	0	5 (29)	1(10)	3 (37)	11 (21)
C (%)	0 (0)	1 (20)	0	7 (41)	2 (20)	1 (13)	11 (21)
D (%)	1 (11)	0 (0)	0	3 (18)	6 (60)	3 (37)	13 (24)

BCLC:Barcelona Clinic Liver Cancer; LT : liver transplantation; LR : liver resection; RABL : radiofrequency ablation; CRE : chemo or radioembolization; SOR : sorafenib; BSC : best supportive care.

**Table 3:** Characteristics at HCC diagnosis of PLWH in the overall sample and according to baseline alphafetoprotein (cut-off = 200 ng/mL).

Characteristics	Category	Overall (*n* = 53)	BL AFP >200 ng/mL (*n* = 15)	BL AFP ≤200 ng/mL (*n* = 38)	*P* value
Age		53 (48–56)	53 (47–55)	53 (50–57)	0.574
Male sex (%)		45 (85)	12 (80)	33 (87)	0.673
Risk factor for HIV (%)	Drug users	26 (49)	9 (60)	17 (45)	0.539
	Sexual exposure	11 (21)	3 (20)	8 (21)	
	Other/Unknown	16 (30)	3 (20)	13 (34)	

Years of HIV infection		24.2 (16.3–27.2)	26.3 (23.2–27.7)	22.1 (15.8–26.4)	0.124
Years of ART		14.7 (9.5–17.8)	15.7 (10.0–17.9)	13.6 (8.8–16.9)	0.435
CD4 nadir cells count, *n*/*μ*L		105 (59–229)	81 (36–162)	122 (65–251)	0.142
CD4 cells count, *n*/*μ*L		392 (222–598)	437 (180–757)	386 (236–548)	0.610
CD8 cells count, *n*/*μ*L		626 (391–1115)	509 (389–1115)	796 (391–1209)	0.556
CD4/CD8 ratio		0.53 (0.34–1.09)	0.75 (0.35–1.20)	0.49 (0.33–0.88)	0.229

Anti-HCV (%)	Positive	36 (68)	13 (87)	23 (61)	0.164
	Negative	14 (26)	2 (13)	12 (32)	
	Unknown	3 (6)	0	3 (8)	

HBsAg	Positive	17 (32)	3 (20)	14 (37)	0.203
	Negative	33 (62)	12 (80)	21 (55)	
	Unknown	3 (6)	0	3 (8)	

Calendar year HCC diagnosis		2012 (2010–2014)	2013 (2010–2015)	2012 (2008–2014)	0.106
AFP, ng/mL		41.8 (15.1–256)	821 (368–3458)	28.3 (11.3–46.0)	<0.0001
AST, IU/L		71 (39–111)	70 (39–93)	72 (34–111)	0.868
ALT, IU/L		67 (31–100)	60 (31–100)	71 (27–100)	0.521
Total bilirubin, mg/dL		1.09 (0.79–1.85)	1.34 (0.99–2.72)	1.04 (0.78–1.75)	0.157

Child–Turcotte–Pugh score	A	40 (75)	12 (80)	28 (74)	0.317
	B	9 (17)	1 (7)	8 (21)	
	C	4 (8)	2 (13)	2 (5)	

Nodules number (%)					0.898
	1	18 (34)	5 (33)	13 (34)	
	2	6 (11)	1 (7)	5 (13)	
	3	4 (8)	1 (7)	3 (8)	
	>3	25 (47)	8 (53)	17 (45)	
				
	<3	24 (45)	6 (40)	18 (47)	
	≥3	29 (55)	9 (60)	20 (53)	

Maximum tumor size (cm)		3.0 (2.0–5.5)	3.0 (2.1–3.4)	2.7 (2.0–7)	0.464
Presence of portal vein thrombosis (%)		19 (36)	6 (40)	13 (34)	0.692

Extrahepatic metastasis (%)	Yes	4 (8)	3 (20)	1 (3)	0.084
	No	48 (91)	12 (80)	36 (95)	
	Unknown	1 (1)	0	1 (3)	

BCLC stage (%)	0	9 (17)	2 (13)	7 (18)	0.948
	A	9 (17)	2 (13)	7 (18)	
	B	11 (21)	3 (20)	8 (21)	
	C	11 (21)	4 (27)	7 (18)	
	D	13 (25)	4 (27)	9 (24)	

HCC treatment (%)	Curative	18 (34)	2 (13)	16 (42)	
	LR	5	1	4	0.046
	LT	9	1	8	
	RABL	4	0	4	
	Non curative	35 (66)	13 (87)	22 (58)	
	CRE	17	6	11	
	SOR	10	3	7	
	BSC	8	4	4	

Results are described by median (IQR) or frequency (%). BL : baseline; AFP : alphafetoprotein; ART : antiretroviral therapy; *n* : number; HBsAg : hepatitis B surface antigen; AST : aspartate aminotransferase (normal values <35 IU/L); ALT : alanine aminotransferase (normal values <59 IU/L); BCLC: Barcelona clinic liver cancer; LR : liver resection; LT : liver transplantation; RABL : radiofrequency ablation; CRE : chemo or radioembolization; SOR : sorafenib; BSC : best supportive care.

**Table 4:** Predictors of death by multivariable Cox proportional-hazards models stratified on two different cut-off (ROC cut-off of 28.8 ng/mL (model 1) and 200 ng/mL (model 2)).

Characteristics at HCC diagnosis	Category	Model 1(N patients=45)		Model 2 (*N* patients=45)	
		aHR (95% CI)	*P* value	aHR (95% CI)	*P* value
Age	Per 3-years older	0.73 (0.51–1.04)	0.081	0.76 (0.56–1.03)	0.079
Sex	Female vs. male	1.05 (0.27–4.08)	0.946	1.14 (0.33–3.90)	0.837
Anti-HCV	Yes vs. no	3.67 (0.62–21.85)	0.153	2.32 (0.39–13.82)	0.357
HBsAg	Yes vs. no	8.57 (1.47–50.08)	0.017	4.89 (0.92–25.98)	0.062
Years of ART	Per 1-year longer	1.23 (0.88–1.72)	0.216	1.28 (0.94–1.75)	0.121
Calendar year of HCC diagnosis	Per 1-more recent year	0.92 (0.48–1.76)	0.798	0.85 (0.47–1.52)	0.579
BCLC stage	Intermediate/advanced vs. very early/early stage	1.26 (0.28–5.76)	0.767	0.86 (0.22–3.35)	0.827
	Terminal vs. very early/early stage	2.38 (0.39–14.38)	0.345	1.08 (0.22–5.22)	0.929
Number of nodules	≥3 vs. <3	1.00 (0.36–2.75)	0.992	1.10 (0.39–3.13)	0.860
AFP levels (ng/mL)	≥28.8 vs <28.2	7.05 (1.94–25.71)	0.003	—	—
	>200 vs ≤200	—	—	3.14 (1.11–8.88)	0.031
CD4+ cell count (number/*µ*L)	Per 100-cells/*µ*L higher	0.91 (0.75–1.11)	0.341	0.97 (0.80–1.17)	0.740
HIV-RNA (copies/mL)	≥50 vs <50	0.52 (0.13–2.10)	0.361	1.48 (0.43–5.05)	0.531
HCC treatment	Curative vs. noncurative	0.08 (0.02–0.40)	0.002	0.06 (0.01–0.29)	0.0004

N : number; HCC : hepatocellular carcinoma; aHR, adjusted hazard ratio; 95% CI, 95% confidence interval; ART : antiretroviral therapy; AIC, Akaike information criteria; HBsAg : hepatitis B surface antigen; BCLC : Barcelona Clinic Liver Cancer; AFP : alpha-phetoprotein.

## Data Availability

The data used to support the findings of this study are available from the corresponding author upon request.

## References

[1] Macías J., Melguizo I., Fernández-Rivera F. (2002). Mortality due to liver failure and impact on survival of hepatitis virus infections in HIV-infected patients receiving potent antiretroviral therapy. *European Journal of Clinical Microbiology & Infectious Diseases*.

[2] Ryom L., Lundgren J. D., De Wit S. (2016). Use of antiretroviral therapy and risk of end-stage liver disease and hepatocellular carcinoma in HIV-positive persons. *AIDS*.

[3] Weber R., Sabin C. A., Friis-Møller N. (2006). Liver-related deaths in persons infected with the human immunodeficiency virus. *Archives of Internal Medicine*.

[4] Tyson G. L., Duan Z., Kramer J. R., Davila J. A., Richardson P. A., El–Serag H. B. (2011). Level of *α*-fetoprotein predicts mortality among patients with hepatitis C-related hepatocellular carcinoma. *Clinical Gastroenterology and Hepatology*.

[5] Kinoshita A., Onoda H., Fushiya N., Koike K., Nishino H., Tajiri H. (2015). Staging systems for hepatocellular carcinoma: current status and future perspectives. *World Journal of Hepatology*.

[6] European Association for the Study of the Liver (2018). Electronic address: easloffice@easloffice.eu; European association for the study of the liver. EASL clinical practice guidelines: management of hepatocellular carcinoma. *Journal of Hepatology*.

[7] Child C. G., Turcotte J. G. (1964). Surgery and portal hypertension. *Major Problems in Clinical Surgery*.

[8] Llovet J., Brú C., Bruix J. (1999). Prognosis of hepatocellular carcinoma: the BCLC staging classification. *Seminars in Liver Disease*.

[9] Chevret S., Trinchet J.-C., Mathieu D., Rached A. A., Beaugrand M., Chastang C. (1999). A new prognostic classification for predicting survival in patients with hepatocellular carcinoma. *Journal of Hepatology*.

[10] The Cancer of the Liver Italian Program (CLIP) Investigators (2000). Prospective validation of the CLIP score: a new prognostic system for patients with cirrhosis and hepatocellular carcinoma. *Hepatology*.

[11] Leung T. W. T., Tang A. M., Zee B. (2002). Construction of the Chinese university prognostic index for hepatocellular carcinoma and comparison with the TNM staging system, the Okuda staging system, and the cancer of the liver Italian program staging system. *Cancer*.

[12] Mitsuhashi N., Kobayashi S., Doki T. (2008). Clinical significance of alpha-fetoprotein: involvement in proliferation, angiogenesis, and apoptosis of hepatocellular carcinoma. *Journal of Gastroenterology and Hepatology*.

[13] Toader E., Bancu A., Mitrică D. E., Constantinescu G., Ştefănescu G., Bălan G. G. (2019). Interrelations between elevated alpha-fetoprotein levels and tumor morphology of patients with hepatocellular carcinoma. *Romanian journal of morphology and embryology = Revue roumaine de morphologie et embryologie*.

[14] Tangkijvanich P., Anukulkarnkusol N., Suwangool P. (2000). Clinical characteristics and prognosis of hepatocellular carcinoma. *Journal of Clinical Gastroenterology*.

[15] Duvoux C., Roudot–Thoraval F., Decaens T. (2012). Liver transplantation for hepatocellular carcinoma: a model including *α*-fetoprotein improves the performance of Milan criteria. *Gastroenterology*.

[16] Pinato D. J., Allara E., Chen T.-Y. (2019). Influence of HIV infection on the natural history of hepatocellular carcinoma: results from a global multicohort study. *Journal of Clinical Oncology*.

[17] Farinati F., Vitale A., Spolverato G. (2016). Development and validation of a new prognostic system for patients with hepatocellular carcinoma. *PLoS Medicine*.

[18] Lida H., Honda M., Kawai H. F. (2005). Ephrin-A1 expression contributes to the malignant characteristics of {alpha}-fetoprotein producing hepatocellular carcinoma. *Gut*.

[19] Li M.-S., Ma Q. L., Chen Q. (2005). Alpha-fetoprotein triggers hepatoma cells escaping from immune surveillance through altering the expression of Fas/FasL and tumor necrosis factor related apoptosis-inducing ligand and its receptor of lymphocytes and liver cancer cells. *World Journal of Gastroenterology*.

[20] Park J. S., Saraf N., Dieterich D. T. (2006). HBV plus HCV, HCV plus HIV, HBV plus HIV. *Current Gastroenterology Reports*.

[21] Hyun L. C. B., Coyle C. W. J. (2004). Hepatocellular carcinoma in a patient with human immunodeficiency virus and hepatitis B virus coinfection: an emerging problem?. *Southern Medical Journal*.

[22] Wallace M. C., Preen D. B., Short M. W., Adams L. A., Jeffrey G. P. (2019). Hepatocellular carcinoma in Australia 1982-2014: increasing incidence and improving survival. *Liver International*.

[23] Lim C., Goutte N., Gervais A. (2012). Standardized care management ensures similar survival rates in HIV-positive and HIV-negative patients with hepatocellular carcinoma. *JAIDS Journal of Acquired Immune Deficiency Syndromes*.

[24] Yau T., Tang V. Y. F., Yao T.-J., Fan S.-T., Lo C.-M., Poon R. T. P. (2014). Development of Hong Kong Liver Cancer staging system with treatment stratification for patients with hepatocellular carcinoma. *Gastroenterology*.

[25] Puoti M., Bruno R., Soriano V. (2004). Hepatocellular carcinoma in HIV-infected patients. *AIDS*.

[26] Merchante N., Rodríguez-Fernández M., Figueruela B. (2020). Impact of HIV on the survival of hepatocellular carcinoma in hepatitis C virus-infected patients. *AIDS*.

[27] Bräu N., Fox R. K., Xiao P. (2007). Presentation and outcome of hepatocellular carcinoma in HIV-infected patients: a U.S.-Canadian multicenter study. *Journal of Hepatology*.

[28] Berretta M., Garlassi E., Cacopardo B. (2011). Hepatocellular carcinoma in HIV-infected patients: check early, treat hard. *The Oncologist*.

[29] Sangiovanni A., Triolo M., Iavarone M. (2018). Multimodality treatment of hepatocellular carcinoma: how field practice complies with international recommendations. *Liver International*.

[30] Guarino M., Tortora R., de Stefano G. (2018). Adherence to Barcelona clinic liver cancer guidelines in field practice: results of progetto epatocarcinoma campania. *Journal of Gastroenterology and Hepatology*.

[31] Granito A., Bolondi L. (2017). Non-transplant therapies for patients with hepatocellular carcinoma and child-pugh-turcotte class B cirrhosis. *The Lancet Oncology*.

[32] Merchante N., Ibarra S., Revollo B. (2017). Real-life experience with sorafenib for the treatment of hepatocellular carcinoma in HIV-infected patients. *AIDS*.

